# Disparities and Factors Associated with Coronavirus Disease-2019-Related Public Stigma: A Cross-Sectional Study in Thailand

**DOI:** 10.3390/ijerph19116436

**Published:** 2022-05-25

**Authors:** Chidchanok Ruengorn, Ratanaporn Awiphan, Chabaphai Phosuya, Yongyuth Ruanta, Kednapa Thavorn, Nahathai Wongpakaran, Tinakon Wongpakaran, Surapon Nochaiwong

**Affiliations:** 1 Department of Pharmaceutical Care, Faculty of Pharmacy, Chiang Mai University, Chiang Mai 50200, Thailand; chidchanok.r@cmu.ac.th (C.R.); ratanaporn.a@cmu.ac.th (R.A.); chaba.pharmacy@gmail.com (C.P.); yongyuth.ruanta@cmu.ac.th (Y.R.); 2 Pharmacoepidemiology and Statistics Research Center (PESRC), Chiang Mai University, Chiang Mai 50200, Thailand; kthavorn@ohri.ca; 3 Ottawa Hospital Research Institute, Ottawa Hospital, Ottawa, ON K1H 8L6, Canada; 4 ICES Ottawa, Ottawa, ON K1Y 4E9, Canada; 5 School of Epidemiology and Public Health, Faculty of Medicine, University of Ottawa, Ottawa, ON K1G 5Z3, Canada; 6 Department of Psychiatry, Faculty of Medicine, Chiang Mai University, Chiang Mai 50200, Thailand; nahathai.wongpakaran@cmu.ac.th (N.W.); tinakon.w@cmu.ac.th (T.W.)

**Keywords:** COVID-19, fear, mental health, perceived risk, public stigma

## Abstract

Coronavirus disease 2019 (COVID-19)-related public stigma is a major challenge, with scarce available evidence. This study aimed to determine the disparities and factors associated with COVID-19-related public stigma in the Thai population. We conducted a cross-sectional study involving a voluntary online survey in Thailand from 21 April 2020 to 4 May 2020. We invited 4004 participants to complete a series of questionnaires, including the validated COVID-19 public stigma scale and questions on relevant COVID-19-related psychosocial issues. Multinomial logistic regression was performed to investigate the factors associated with COVID-19-related public stigma. The prevalence of COVID-19-related public stigma was 24.2% (95% confidence interval [CI], 22.2–26.2) for no/minimal, 35.5% (95% CI, 33.4–37.6) for moderate, and 40.3% (95% CI, 38.2–42.4) for high. We observed disparities in the prevalence of COVID-19-related public stigma according to participant characteristics and psychosocial factors. Using the no/minimal group as a reference group, the six predominant risk factors significantly associated with a moderate and high degree of COVID-19-related public stigma were middle-aged or older adults, male, divorced/widowed/separated, current quarantine status, moderate/severe fear of COVID-19, and medium/high perceived risk of COVID-19. Additional risk factors significantly related to a high degree of COVID-19-related public stigma were religion (Buddhist), region of residence (non-capital city), and exposure to COVID-19-related information. Disparities in COVID-19-related public stigma due to sociodemographic and psychosocial issues are frequent in the Thai population. To reduce public stigmatization, early identification of vulnerable groups and the development of tailored mitigation strategies should be implemented during the pandemic.

## 1. Introduction

The incidence of psychosomatic illness has increased since the emergence of coronavirus disease 2019 (COVID-19). These conditions are expected natural psychological responses to unpredictable, fast-spreading infectious diseases similar to conditions experienced during prior outbreaks such as SAR-CoV or MER-CoV [1]. Measures to contain the spread of the virus, such as lockdowns, home confinement strategies, restriction of travelling, and misinformation obtained from online social networking sites, have been shown to be detrimental [1,2].

COVID-19 has caused universal awareness, anxiety, and distress, partly due to fear of infection, leading to the so-called COVID-19 effect [3,4]. The COVID-19 effect provokes disease-associated social stigma, xenophobia, and discrimination against people who are perceived to have been in contact with the virus or those with certain ethnic backgrounds [3]. Social stigma or public stigma relating to infectious diseases has long been acknowledged in the past, such as that for HIV, hepatitis C virus, tuberculosis, and Zika, and is now acknowledged amid the COVID-19 pandemic [5]. Public stigma usually creates discriminatory behaviors, such as isolation, refusal to receive services, harassment, and bullying. People who are victims of social stigma can develop social avoidance, denial of healthcare, and perhaps even be in danger of violence [1]. The incidence of hate crime towards specific ethnicities (i.e., Asians) has been reported in the United States and worldwide. In addition, stigma toward COVID-19 may lead to adverse mental health outcomes, including suicidal behavior [6,7].

To date, few studies have reported the prevalence and factors associated with COVID-19-related public stigma. None of the existing studies have focused on public stigma in Thailand. While the pandemic is still ongoing, understanding COVID-19-related public stigma and its related factors can help define the target population prone to social stigma and develop tailored mitigation strategies. Therefore, we conducted this study to determine the prevalence of, and factors associated, with COVID-19-related public stigma in the Thai population.

## 2. Materials and Methods

### 2.1. Study Design and Participants

This was a cross-sectional analytical study based on the Health Outcomes and Mental Health Care Evaluation Survey, under the Pandemic Situation of COVID-19 (HOME-COVID-19). The details of the protocol have been published elsewhere [8]. In brief, an open, online, voluntary survey encompassing a set of questionnaires was sent via the SurveyMonkey^®^ platform, which limits to one-time participation per unique Internet Protocol address. The samples were invited by a convenience and snowball sampling strategy from all the regions in Thailand through various social media networks including public websites, Facebook, LINE, Twitter, and Instagram. Eligible participants included (i) Thai citizens aged ≥18 years at the date of the survey, (ii) permanent residents or non-residents with work permits, (iii) those who could read and communicate in the Thai language, and (iv) those who could access the Internet. We excluded incomplete surveys and surveys that took <2 min or >60 min to complete. The current analysis was restricted to only wave I of information from 21 April 2020 to 4 May 2020 (during the national government’s protocols under lockdown in Thailand).

Under the HOME-COVID-19, this current study was approved by the Committee of Research Ethics of the Faculty of Public Health (ET010/2020) and the Faculty of Pharmacy (23/2563), Chiang Mai University. All participants provided written informed consent for the first page of the questionnaire. This study was in line with the Strengthening the Reporting of Observational Studies in Epidemiology Statement [9] and Improving the Quality of Web Surveys: The Checklist for Reporting Results of Internet E-Surveys [10].

### 2.2. Sample Size

The sample sizes for both prevalence and factors related to public stigma in the Thai population were estimated using (i) the overall mean ± standard deviation (SD) of COVID-19-related public stigma (based on the validated COVID-19 Public Stigma Scale (COVIDSS)) of 24.2 ± 7.6 [11], specified type I error at 0.05, and d equal to 0.5, a total sample size of 891; and (ii) linear multiple regression, *R*^2^ deviation from zero with a small effect size of 0.02, type I error of 0.05, 90% of power, and anticipated total predictors equal to 15, with a sample size needed of 1192 [12]. The sample size of our survey met both requirements and was sufficient to address the research questions.

### 2.3. Assessment Tools and Potential Risk Factors

Participants were asked to complete a set of questionnaires regarding COVID-19-related public stigma and relevant psychosocial issues as follows:

- Public stigma: COVID-PSS comprises ten items with three factors (stereotypes, prejudice, fear), and a possible score range of 10-50 points. The COVID-PSS revealed acceptable psychometric properties in the Thai population, with Cronbach’s α of 0.85. The degree of public stigma was established and classified as no/minimal (≤18 points), moderate (19–25 points), or high (≥26 points) [11].
- Perceived social support: The Multidimensional Scale of Perceived Social Support (MSPSS-12) consists of 12 items that measure individual perceptions of external social support. This scale has excellent internal consistency, with a Cronbach’s α of 0.92 [13]. Perceived social support was categorized as low (12–35 points), moderate (36–60 points), or high (61–84 points).
- Resilient coping: The Brief Resilient Coping Scale (BRCS) consists of four items to capture tendencies to cope with stress in a highly adaptive manner. The BRCS revealed satisfactory reliability (Cronbach’s α = 0.80) [8]. For scale interpretation, BRCS scores was classified as low- (≤13 points), medium- (14–16 points), or high (≥17 points) resilience copers [14].
- Fear of COVID-19 and perceived risk of COVID-19 infection: A numerical rating scale (NRS) of 0–10 points was used to measure the degree of fear or perceived risk of COVID-19 infection. The degree of fear or perceived risk was classified as no/minimal fear or low perceived risk (0–3 points), moderate fear or medium perceived risk (4–6 points), and severe fear or high perceived risk (7–10 points).
- A set of potential risk factors for public stigma, including sociodemographic characteristics (age, sexual identity, marital status, educational level, occupation, religion, region of residence, living status, personal income, reimbursement scheme, history of mental illness, chronic non-communicable diseases (NCDs)), and issues-related to the COVID-19 pandemic (economic burden (income loss, financial problems), duration of exposure to COVID-19-related information, confirmed cases in the community, quarantine status, and working from home status).

### 2.4. Statistical Analyses

All analyses were performed using Stata 14.0 (StataCorp, LP, College Station, TX, USA). Two-tailed tests were conducted with a type I error rate of 0.05. Respondents with missing data or incomplete data were excluded from the analysis. Descriptive statistics are expressed as frequency and percentage or mean ± SD, with a range (min-max). We categorized COVID-19-related public stigma into three groups according to total scores (no/minimal, moderate, and high degrees of stigma). Baseline participant characteristics, according to the degree of public stigma, were assessed using analysis of covariance for continuous data and Fisher’s exact test for categorical data. We applied survey weights to all analyses to ensure that our results represented the national population and rate of Internet use based on the National Statistic Office of the Thai Ministry of Information and Communication Technology.

We estimated the prevalence rate of COVID-19-related public stigma with 95% confidence intervals (CIs) and assessed the variation in these rates by participant characteristics. Using a trend test analysis, non-overlapping 95% CIs (*p* for trend <0.05) indicated a statistical difference in the prevalence rates across participant characteristic strata. We applied a two-stage multinomial logistic regression approach to determine factors associated with the degree of COVID-19-related public stigma (using no/minimal as a reference group). In the first stage, the crude association between participant characteristics and the degree of public stigma (moderate or high) was analyzed using univariable multinomial logistic regression models to identify candidate risk factors. Next, candidate risk factors with a *p*-value < 0.200 were included in the multivariable multinomial logistic regression models using the stepwise backward method. Variance inflation factors were used to identify multicollinearity in the final model. Moreover, an ancillary analysis was performed to confirm the robustness of the main analysis using multivariable linear regression to explore the linear relationship between the potential risk factors and COVID-PSS—public stigma score. The effect estimates of the risk factor models are expressed as odds ratios (ORs) or beta coefficients with 95% CIs.

## 3. Results

### 3.1. Overview of Participant Characteristics

**Figure 1:**
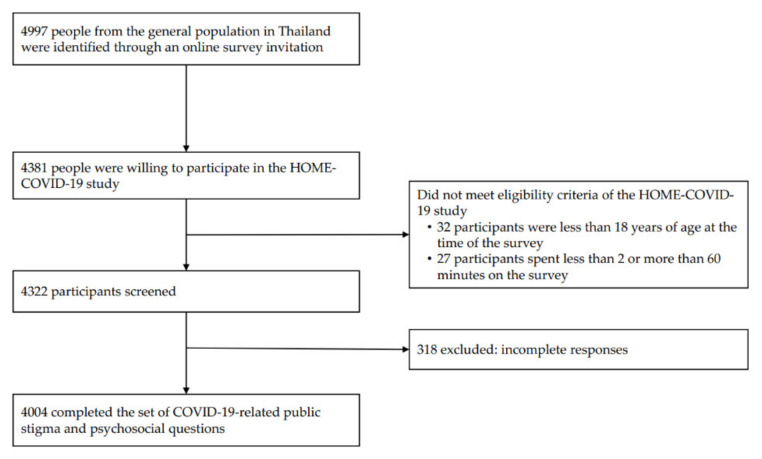
Flow diagram for study participants. Abbreviation: HOME-COVID-19, The Health Outcomes and Mental Health Care Evaluation Survey Research Group-Coronavirus disease 2019.

A total of 4997 people were identified through an online survey invitation. Of those, 4381 (87.7%) were willing to participate in the survey, and 4004 participants met the eligibility criteria and completed a set of mental health and psychosocial questions (completeness rate of 92.6%, Figure 1). Participant characteristics are described in Supplementary Table S1. Most participants were female (65.4%), with a mean age of 29.1 ± 10.8 years (range 18–79). Most participants resided in a non-capital city or its environs (64.4%). Most participants had moderate/high perceived social support and were medium/high resilient copers, whereas most participants reported having moderate/severe fear of COVID-19 and medium/high perceived risk of COVID-19 infection.

### 3.2. Prevalence and Disparities of COVID-19-Related Public Stigma

The overall mean COVID-PSS—public stigma score was 24.2 ± 7.6 (range 10–50). With respect to the degree of COVID-19-related public stigma, the unadjusted prevalence estimate was 24.2% (95% CI, 22.2–26.2) for no/minimal, 35.5% (95% CI, 33.4–37.6) for moderate, and 40.3% (95% CI, 38.2–42.4) for high. Remarkably, statistical differences in the prevalence rates across participants with a high degree of COVID-19-related public stigma were observed, particularly in participants who had a high perceived risk of COVID-19 infection (82.3%; 95% CI, 79.1–85.2), followed by participants aged ≥51 years (66.8%; 95% CI, 58.0–74.5) and participants who had a severe fear of COVID-19 (63.0%; 95% CI, 60.0–65.9) (Table 1). Moreover, participants’ age, sexual identity, marital status, religion, region of residence, reimbursement scheme, history of NCDs, information exposure during the COVID-19 pandemic, confirmed cases in the community, quarantine status, perceived social support, fear of COVID-19, and perceived risk of COVID-19 infection were associated with prevalence across the degree of COVID-19-related public stigma (*p* for trend <0.05, Table 1).

### 3.3. Risk Factors Associated with COVID-19-Related Public Stigma

With respect to participant characteristics (using no/minimal public stigma as a reference group), the univariable multinomial regression identified 16 candidate risk factors with *p*-value < 0.200 (Table 2). Subsequently, the final model based on multivariable multinomial regression models revealed six independent significant risk factors of moderate degree of COVID-19-related public stigma: (i) ages 31–50 years (adjusted OR, 1.59; 95% CI, 1.08–2.34) and ≥51 years (adjusted OR, 4.34; 95% CI, 1.49–12.64), (ii) male sex (adjusted OR, 1.68; 95% CI, 1.21–2.33), (iii) divorced/widowed/separated (adjusted OR, 3.88; 95% CI, 1.42–10.55), (iv) current quarantine status (adjusted OR, 2.08; 95% CI, 1.31–3.33), (v) moderate fear of COVID-19 (adjusted OR, 3.70; 95% CI, 1.95–7.03) and severe fear of COVID-19 (adjusted OR, 6.24; 95% CI, 3.16–12.36), and (vi) medium perceived risk of COVID-19 infection (adjusted OR, 7.78; 95% CI, 5.61–10.79) and high perceived risk of COVID-19 infection (adjusted OR, 41.94; 95% CI, 17.56–100.15) (Table 2).

Meanwhile, the multivariable multinomial regression models recognized nine independent significant risk factors for high degree COVID-19-related public stigma: (i) aged 31–50 years (adjusted OR, 2.22; 95% CI, 1.36–3.62) and ≥51 years (adjusted OR, 10.31; 95% CI, 3.13–34.01), (ii) male sex (adjusted OR, 2.35; 95% CI, 1.58–3.49), (iii) marital status—married/domestic partnership (adjusted OR, 1.97; 95% CI, 1.07–3.64) and divorced/widowed/separated (adjusted OR, 3.69; 95% CI, 1.00–13.60), (iv) religion—Buddhism (adjusted OR, 2.11; 95% CI, 1.22–3.63), (v) non-capital city and its environs (adjusted OR, 1.45; 95% CI, 1.08–1.95), (vi) exposure to COVID-19-related information 1–2 h/day (adjusted OR, 1.52; 95% CI, 1.05–2.21), (vii) current quarantine status (adjusted OR, 1.75; 95% CI, 1.03–2.97), (viii) moderate fear of COVID-19 (adjusted OR, 20.71; 95% CI, 4.11–104.19) and severe fear of COVID-19 (adjusted OR, 111.70; 95% CI, 21.90–569.63), and (ix) medium perceived risk of COVID-19 infection (adjusted OR, 60.15; 95% CI, 23.80–152.00) and high perceived risk of COVID-19 infection (adjusted OR, 2245.43; 95% CI, 667.25–7556.23) (Table 2).

With respect to the ancillary analysis, the findings showed consistent results for the set of factors associated with COVID-19-related public stigma (R^2^ = 0.58), except for exposure to COVID-19-related information, which was not significant. On the other hand, resilient coping perception has become a significant protective factor for COVID-19-related public stigma, with a small effect size (beta coefficient of −0.76; 95% CI, −1.46 to −0.05; *p* = 0.036 for medium resilient copers; −0.82, 95% CI, −1.51 to −0.14; *p* = 0.019 for high resilient copers; Supplementary Table S2).

## 4. Discussion

Our findings highlight the prevalence and disparities of COVID-19-related public stigma in the general Thai population. We found that COVID-19-related public stigma was common during the pandemic in Thailand. Critically, the estimated prevalence of a high degree of COVID-19-related public stigma was frequent at 40.3% (95% CI, 38.2–42.4); this rate was highly variable by participant characteristics and psychosocial issues regarding the pandemic. Moreover, our risks set findings for the development of a medium/high degree of COVID-19-related public stigma can provide information on the target-specific population and minimize public stigmatization in public health settings.

Few studies have addressed COVID-19-related public stigma in the general population owing to the lack of a validated tool. These studies have shown that COVID-19-related public stigma is a common phenomenon in several countries [15,16,17,18,19,20]. Similar to our study, the prevalence of moderate and high degrees of COVID-19-related public stigma accounts for more than half of the surveyed population in many countries. Furthermore, our findings underscore that participant characteristics as well as psychosocial issues during the pandemic are significantly associated with the degree of COVID-19-related public stigma.

Collectively, based on common risk factors, our findings revealed that middle-aged adults or older (31–50, ≥51 years) had a higher risk of being at a moderate/high degree of COVID-19-related public stigma than young adults. Not surprisingly, the older population, particularly in advanced age and with multi-morbidities, is at risk of a severe and critical stage if infected with COVID-19, leading to greater awareness of infection. Males have also been reported to have more severe COVID-19 [21], which may be recognized as at risk of experiencing moderate/high COVID-19-related public stigma than others. Interestingly, the explanation may be related to ideas of masculinity, in which norms are social rules that expect men to be strong, and may invoke behaviors showing responsibility towards protecting their family and community [22]. According to previous reports [23,24], we found that cohabitants are more aware of COVID-19 infection due to fear of infecting their partners. Additionally, married people, particularly healthcare workers, illustrated more personalized stigma and had concerns about public attitudes [24]. People who experienced the current quarantine status certainly perceived a higher risk of COVID-19 infection and had a higher stigma score in our observation. Some studies have shown that quarantine cases are prone to self-stigma and stigmatization by society [23]. In addition, a study involving quarantined healthcare workers also reported that guilt towards family members and friends leads to avoiding contact with neighbors and the community [25]. Finally, both perceived fear and risk of COVID-19 infection were recognized as strong factors contributing to COVID-19-related public stigma in our study. Indeed, the feeling of fear and subsequently the perceived risk of a newly emerging infectious disease usually arises from the uncertain and unpredictable course of the disease. Technology, including the internet and social media, creates “infodemics” spreading the news of COVID-19 cases, mortality, and its communicability. This can accelerate more fear and perceived dangerousness to people. Moreover, stigma from perceived risk can also be mediated by fear of COVID-19.

Apart from the common factors, some unique variables, including religion—Buddhism, living in a non-capital city, and exposure to COVID-19-related information, are associated only with a high degree of COVID-19-related public stigma. In Thailand, Buddhism is the religion of most of the population (93.5%), followed by Muslims (5.4%), and Christians and others (1.1%) [26]. Buddhists are associated with the doctrine of cultivating compassion to attenuate prejudiced attitudes towards other social groups [27]. In the case of the COVID-19 pandemic, comparison with the irreligious population may reflect aspects including liberality and acceptance of the behaviors of others, including mistakes or errors. However, some hidden residual factors, such as borderline personality disorder, narcissism, or carelessness towards religion, predominantly in the younger population, which are associated with lower stigmatization [28,29], were not investigated in our study. Therefore, this finding requires further confirmation. For residential areas, we postulated that people living in the non-capital area had a higher degree of COVID-19-related public stigma because of their fear and perceived risk of COVID-19 infection spreading from the capital city, because most cases in Thailand at the time of data collection were based in the capital city and its environs. Theoretically, media exposure has been suggested as a potential factor for perceived stigmatization among people at a high risk of contagion [30]. In this case, we can determine that media images and influences may lead to prejudice and discrimination related to COVID-19, resulting in violence against some ethnic groups, such as Asian people in the US and globally.

### 4.1. Strengths and Limitations

To our knowledge, this is the first study to report on the prevalence of COVID-19-related public stigma in Thailand. This study was based on a nationwide survey with a large sample size. Unlike previous studies, we used the validated COVID-PSS to measure COVID-19-related public stigma among the Thai population [11]. However, our results should be used with consideration of some limitations. First, we used Wave I of the HOME-COVID-19, representing only the early phase of the pandemic when circumstances could be different from other periods, for example, due to the availability of the COVID-vaccine. We also lacked information regarding knowledge of COVID-19 infection, in which misinformation or lack of knowledge may generate more fear and anxiety about the disease and increase stigma. Although stigma changes over time and context, the results of this study are believed to be beneficial for future emerging infectious diseases. Second, our findings were based on an open online survey; therefore, information bias should be considered. In addition, it may be generalized only to those with access to the Internet. Third, fear of COVID-19 and perceived risk of COVID-19 infection was assessed via a non-validated NRS of 0–10 points questionnaire which may limit comparison to other settings, or international comparisons with respect to these issues. However, NRS—unidimensional assessment is practical, reasonable, and applicable for capturing participants’ feelings or opinions via a public survey. Fourth, despite an ancillary analysis confirming risk factors in line with the main analysis, uncertainty with respect to exposure to COVID-19-related information, resilient coping perception, and the risk of COVID-19-related public stigma need to be confirmed in further studies. Fifth, further associations between public stigma and adverse mental health (i.e., anxiety, stress, and depression) are warranted to address public health concerns. Lastly, the longer-term effect of stigma research needs to be studied, because our findings reflected only the short-term effects, and the impact of stigma may change as the pandemic evolves.

### 4.2. Implications for Public and Future Research

Given the high burden of mental health and psychosocial issues during the pandemic, it is crucial to minimize COVID-19-related public stigma due to the negative consequences of stigma, including an unwillingness to disclose COVID-19 infection or to test and seek treatment. Recently, a randomized trial in the general United States population by [31] suggested that video-based interventions involving reliable information on COVID-19 prevention strategies, video encouraging digital social activity, and video sensitizing to COVID-19-related stigma are effective in reducing COVID-19-related public stigma. However, its utility could be limited by its generalizability to other populations and cross-cultural adaptation to larger public health effects. To help the target population and supplement the previous intervention trial, our findings support proactive mental health surveillance by identifying the person who may be vulnerable or at risk of public stigmatization middle-aged adults or older, male sex, married/domestic partnership, divorced/widowed/separated, Buddhist, living in the non-capital city, current quarantine status, high perceived fear or risk of COVID-19 infection. To promote mental health well-being, multimodal mitigation strategies involving public health education and knowledge, programs for empowering and supporting vulnerable populations, and anti-stigma policies enforced in legal legislation should be promptly implemented during the pandemic.

## 5. Conclusions

COVID-19-related public stigma is highly prevalent and varies among the Thai populations. The results of this study highlight the disparities in the prevalence of COVID-19-related public stigma according to sociodemographic and psychosocial issues. Our study also shows the possibility of identifying vulnerable groups and participants who are at risk of stigma during the pandemic, which should be targeted by strategies aimed at mitigating the impact of public stigma on health.

## Figures and Tables

**Table 1:** Prevalence of COVID-19-related public stigma among general population in Thailand.

Participant Characteristics	Degree of COVID-19-Related Public Stigma								
	No/Minimal: COVID-PSS ≤ 18 Points (n = 983)			Moderate: COVID-PSS 19–25 Points (n = 1364)			High: COVID-PSS ≥ 26 Points (n = 1657)		
	No. of Cases/No. of Total	Prevalence Estimated (% [95% CI]) ^†^	*p*-Value	No. of Cases/No. of Total	Prevalence Estimated (% [95% CI]) ^†^	*p*-Value	No. of Cases/No. of Total	Prevalence Estimated (% [95% CI]) ^†^	*p*-Value
Age, year									
≤30	704/2659	25.9 (23.7–28.4)	<0.001	953/2659	37.1 (34.5–39.8)	<0.001	1002/2659	37.0 (34.4–39.6)	<0.001
31–50	253/1088	23.8 (20.3–27.7)		355/1088	33.6 (29.9–37.5)		480/1088	42.6 (38.7–46.7)	
≥51	26/257	8.0 (4.6–13.6)		56/257	25.2 (18.2–33.8)		175/257	66.8 (58.0–74.5)	
Sexual identity									
Female	673/2619	25.5 (23.3–28.0)	<0.001	896/2619	35.4 (32.8–38.0)	0.845	1050/2619	39.1 (36.5–41.8)	0.227
Male	260/1231	19.3 (16.4–22.6)		415/1231	36.0 (32.3–39.9)		556/1231	44.7 (40.8–48.6)	
Others	50/154	34.5 (24.3–46.4)		53/154	34.1 (24.2–45.7)		51/154	31.4 (21.7–43.0)	
Marital status									
Single	854/3208	26.2 (24.1–28.4)	<0.001	1162/3208	37.7 (35.4–40.2)	<0.001	1192/3208	36.1 (33.8–38.5)	<0.001
Married/domestic partnership	115/693	17.6 (13.8–22.2)		170/693	23.6 (19.6–28.1)		408/693	58.8 (53.6–63.8)	
Divorced/widowed/separated	14/103	5.6 (2.9–10.7)		32/103	42.6 (29.4–57.0)		57/103	51.7 (38.0–65.2)	
Education level									
Illiterate/primary school/junior high school	28/127	21.0 (12.1–33.9)	0.474	41/127	35.6 (24.9–47.9)	0.784	58/127	43.4 (32.0–55.6)	0.373
Senior high school/diploma/high vocational	482/1893	25.4 (22.7–28.2)		654/1893	36.4 (33.4–39.5)		757/1893	38.2 (35.2–41.4)	
Bachelor’s degree/ higher education	473/1984	23.1 (20.6–25.8)		669/1984	34.4 (31.5–37.4)		842/1984	42.5 (39.4–45.6)	
Occupation									
Unemployed/retried	95/391	23.4 (18.3–29.5)	0.276	129/391	38.1 (31.3–45.5)	0.112	167/391	38.4 (31.9–45.3)	0.074
Employed	480/2024	24.2 (21.6–27.0)		663/2024	32.4 (29.7–35.3)		881/2024	43.3 (40.3–46.4)	
College student	408/1589	24.4 (21.6–27.5)		572/1589	38.0 (34.7–41.5)		609/1589	37.5 (34.2–41.0)	
Religion									
Irreligion	143/375	32.9 (26.8–39.6)	0.167	126/375	39.1 (32.2–46.6)	0.795	106/375	28.0 (22.0–34.9)	<0.001
Buddhist	787/3454	35.3 (33.1–37.6)		1183/3454	35.3 (33.1–37.6)		1484/3454	41.8 (39.4–44.1)	
Christian/Muslim/Others	53/175	28.8 (21.1–38.1)		55/175	29.6 (21.4–39.4)		67/175	41.6 (32.6–51.2)	
Region of residence									
Capital city and its environs	412/1425	28.9 (26.6–31.3)	<0.001	498/1425	34.9 (32.5–37.5)	0.382	515/1425	36.1 (33.7–38.7)	<0.001
Non-capital city and its environs	571/2579	22.9 (20.6–25.3)		866/2579	35.6 (33.0–38.3)		1142/2579	41.5 (38.8–44.2)	
Living status									
Alone	161/576	27.0 (22.0–32.7)	0.547	203/576	36.7 (31.2–42.5)	0.473	212/576	36.3 (30.7–42.3)	0.224
With family	745/3164	23.4 (21.4–25.6)		1074/3164	35.4 (33.0–37.8)		1345/3164	41.2 (38.8–43.6)	
With others	77/264	29.8 (22.3–38.6)		87/264	34.5 (26.4–43.6)		100/264	35.6 (27.4–44.8)	
Person income, baht/month ^§^									
≤10,000 (≤308 USD)	465/1905	24.8 (22.1–27.7)	0.124	654/1905	36.2 (33.1–39.4)	0.747	786/1905	39.0 (35.9–42.2)	0.100
10,001–20,000 (309–616 USD)	299/1054	28.0 (24.3–32.0)		357/1054	35.7 (31.8–39.8)		398/1054	36.3 (32.3–40.5)	
>20,000 (>616 USD)	219/1045	19.0 (16.1–22.3)		353/1045	33.7 (30.0–37.6)		473/1045	47.3 (43.2–51.4)	
Reimbursement scheme									
Government/state enterprises	112/539	20.3 (15.9–25.5)	0.404	157/539	30.7 (25.4–36.5)	0.116	270/539	49.0 (43.1–55.0)	0.025
Universal coverage scheme	346/1329	26.1 (22.8–29.7)		466/1329	36.6 (32.9–40.5)		517/1329	37.3 (33.6–41.1)	
Social security scheme	284/1161	23.8 (20.6–27.4)		402/1161	36.3 (32.6–40.1)		475/1161	39.9 (36.1–43.8)	
Self-payment/others	241/975	24.7 (21.2–28.6)		339/975	36.3 (32.3–40.6)		395/975	39.0 (34.9–43.2)	
History of mental illness									
No	875/3645	23.7 (21.8–25.7)	0.527	1249/3645	35.5 (33.3–37.7)	0.394	1521/3645	40.8 (38.6–43.1)	0.158
Yes	108/359	30.3 (24.2–37.2)		115/359	35.4 (28.6–42.7)		136/359	34.4 (28.0–41.3)	
History of chronic NCDs ^‡^									
No	861/3405	25.2 (23.2–27.3)	0.010	1187/3405	36.2 (34.0–38.6)	0.114	1357/3405	38.6 (36.3–40.9)	<0.001
Yes	122/599	18.4 (14.6–23.1)		177/599	30.9 (25.9–36.4)		300/599	50.6 (44.9–56.3)	
Income loss during the COVID-19 pandemic									
No	585/2340	23.9 (21.5–26.4)	0.433	825/2340	36.2 (33.5–39.0)	0.060	930/2340	39.9 (37.1–42.7)	0.125
Yes	398/1664	24.8 (21.9–27.9)		539/1664	34.4 (31.2–37.7)		727/1664	40.8 (37.6–44.2)	
Financial problems during the COVID-19 pandemic									
No	498/1992	24.4 (21.8–27.1)	0.511	713/1992	37.3 (34.3–40.3)	0.218	781/1992	38.3 (35.4–41.4)	0.540
Yes	485/2012	23.1 (21.5–26.9)		651/2012	33.8 (30.9–36.8)		876/2012	42.1 (39.1–45.2)	
Information exposure during the COVID-19 pandemic									
<1 h/day	408/1481	27.0 (23.9–30.4)	<0.001	503/1481	35.5 (32.1–39.1)	0.726	570/1481	37.4 (34.0–40.9)	0.128
1–2 h/day	391/1644	22.9 (20.2–25.9)		571/1644	35.9 (32.7–39.3)		682/1644	41.1 (37.8–44.5)	
≥3 h/day	184/879	43.5 (38.9–48.3)		290/879	34.6 (30.2–39.2)		405/879	43.5 (38.9–48.3)	
Confirmed cases in the community									
No	637/2562	25.1 (22.7–27.7)	0.808	871/2562	35.2 (32.5–38.0)	0.775	1054/2562	39.7 (37.0–42.5)	0.025
Yes	136/641	20.2 (16.5–24.6)		215/641	33.1 (28.6–37.9)		290/641	46.6 (41.7–51.6)	
Not known	210/801	25.1 (21.4–29.2)		278/801	38.7 (34.1–43.4)		313/801	36.3 (31.9–40.9)	
Quarantine status									
Never	486/1781	27.6 (24.6–30.7)	<0.001	567/1781	32.3 (29.3–35.5)	0.059	728/1781	40.1 (36.8–43.4)	0.456
Past	359/1575	22.6 (19.9–25.6)		563/1575	36.5 (33.2–39.8)		653/1575	40.9 (37.6–44.3)	
Current	138/648	20.1 (16.2–24.5)		234/648	40.8 (35.4–46.3)		276/648	39.2 (34.1–44.6)	
Working from home									
No	209/865	23.9 (20.1–28.1)	0.764	293/865	34.1 (29.9–38.5)	0.892	363/865	42.1 (37.6–46.7)	0.695
Yes	774/3139	24.3 (22.2–26.5)		1071/3139	35.8 (33.5–38.3)		1294/3139	39.8 (37.4–42.3)	
Perceived social support									
Low perceived support	59/226	32.9 (24.6–42.6)	<0.001	69/226	33.7 (25.8–42.6)	0.137	98/226	33.4 (26.3–41.3)	0.653
Moderate perceived support	501/1833	27.1 (24.4–30.1)		574/1833	32.7 (29.7–35.8)		758/1833	40.2 (37.1–43.4)	
High perceived support	423/1945	20.5 (18.1–23.2)		721/1945	38.4 (35.3–41.6)		801/1945	41.1 (38.0–44.3)	
Resilient coping									
Low resilient copers	425/1756	23.9 (21.2–26.8)	0.815	605/1756	36.8 (33.7–40.1)	0.864	726/1756	39.3 (36.2–42.4)	0.969
Medium resilient copers	393/1570	25.2 (22.1–28.5)		525/1570	34.2 (30.9–37.6)		652/1570	40.6 (37.2–44.2)	
High resilient copers	165/678	23.1 (18.9–27.8)		234/678	34.7 (29.7–40.1)		279/678	42.2 (36.9–47.8)	
Fear of COVID-19									
No/minimal	169/200	82.9 (74.2–89.0)	<0.001	29/200	16.4 (10.3–25.1)	<0.001	2/200	0.7 (0.2–2.9)	<0.001
Moderate	662/1698	38.7 (35.4–42.0)		754/1698	45.8 (42.4–49.2)		282/1698	15.5 (13.3–18.1)	
Severe	152/2106	8.1 (6.5–9.9)		581/2106	28.9 (26.2–31.8)		1373/2106	63.0 (60.0–65.9)	
Perceived risk of COVID-19 infection									
Low perceived risk	584/767	74.5 (69.8–78.7)	<0.001	171/767	23.8 (19.7–28.5)	<0.001	12/767	1.7 (0.8–3.7)	<0.001
Medium perceived risk	385/1997	19.9 (17.5–22.5)		990/1997	51.6 (48.5–54.6)		622/1997	28.6 (25.9–31.4)	
High perceived risk	14/1240	1.0 (0.4–2.2)		203/1240	16.7 (13.9–19.9)		1023/1240	82.3 (79.1–85.2)	
Overall	983/4004	24.2 (22.2–26.2)		1364/4004	35.5 (33.4–37.6)		1657/4004	40.3 (38.2–42.4)	

^†^ Prevalence is presented as weighted. ^‡^ To includes diabetes mellitus, hypertension, dyslipidemia, stroke and heart disease, chronic kidney disease, chronic lung disease, and cancer. ^§^ The currency exchange on the survey period was 1 USD = 32.5 Baht. Abbreviations: CI, confidence interval; COVID-19, coronavirus disease-2019; COVID-PSS, coronavirus disease-2019 Public Stigma Scale; NCDs, non-communicable diseases.

**Table 2:** Multinomial logistic regression model results of factors associated with COVID-19-related public stigma (n = 4004).

Factors	Moderate vs. No/Minimal				High vs. No/Minimal			
	Unadjusted OR (95% CI) ^†^	*p*-Value	Adjusted OR (95% CI) ^†^	*p*-Value	Unadjusted OR (95% CI) ^†^	*p*-Value	Adjusted OR (95% CI) ^†^	*p*-Value
Age, year								
≤30	Reference (1.00)		Reference (1.00)		Reference (1.00)		Reference (1.00)	
31–50	0.99 (0.76–1.29)	0.927	1.59 (1.08–2.34)	0.018	1.26 (0.97–1.63)	0.083	2.22 (1.36–3.62)	0.001
≥51	2.19 (1.11–4.35)	0.024	4.34 (1.49–12.64)	0.007	5.84 (3.14–10.83)	<0.001	10.31 (3.13–34.01)	<0.001
Sexual identity								
Female	Reference (1.00)		Reference (1.00)		Reference (1.00)		Reference (1.00)	
Male	1.34 (1.03–1.75)	0.028	1.68 (1.21–2.33)	0.002	1.51 (1.17–1.95)	0.001	2.35 (1.58–3.49)	<0.001
Others	0.72 (0.40–1.28)	0.256	1.00 (0.50–2.03)	0.992	0.59 (0.33–1.08)	0.088	1.00 (0.42–2.37)	0.993
Marital status								
Single	Reference (1.00)		Reference (1.00)		Reference (1.00)		Reference (1.00)	
Married/domestic partnership	0.93 (0.65–1.33)	0.690	0.88 (0.52–1.49)	0.627	2.42 (1.75–3.36)	<0.001	1.97 (1.07–3.64)	0.030
Divorced/widowed/separated	5.24 (2.36–11.64)	<0.001	3.88 (1.42–10.55)	0.008	6.64 (3.16–13.99)	<0.001	3.69 (1.00–13.60)	0.050
Education level								
Illiterate/primary school/junior high school	Reference (1.00)				Reference (1.00)			
Senior high school/diploma/high vocational	0.85 (0.40–1.79)	0.655			0.73 (0.36–1.49)	0.387		
Bachelor’s degree/higher education	0.88 (0.42–1.85)	0.736			0.89 (0.44–1.82)	0.748		
Occupation								
Unemployed/retried	Reference (1.00)				Reference (1.00)			
Employed	0.82 (0.55–1.23)	0.340			1.09 (0.75–1.60)	0.645		
College student	0.96 (0.64–1.44)	0.833			0.94 (0.64–1.38)	0.747		
Religion								
Irreligion	Reference (1.00)				Reference (1.00)		Reference (1.00)	
Buddhist	1.29 (0.90–1.85)	0.159			2.14 (1.47–3.11)	<0.001	2.11 (1.22–3.63)	0.007
Christian/Muslim/Others	0.86 (0.47–1.59)	0.632			1.69 (0.95–3.01)	0.073	1.41 (0.59–3.35)	0.439
Region of residence								
Capital city and its environs	Reference (1.00)				Reference (1.00)			
Non-capital city and its environs	1.29 (1.06–1.57)	0.012			1.45 (1.20–1.76)	<0.001	1.45 (1.08–1.95)	0.013
Living status								
Alone	Reference (1.00)				Reference (1.00)			
With family	1.11 (0.80–1.54)	0.523			1.31 (0.94–1.83)	0.114		
With others	0.85 (0.50–1.46)	0.564			0.89 (0.52–1.54)	0.676		
Person income, baht/month ^§^								
≤10,000 (≤308 USD)	Reference (1.00)				Reference (1.00)			
10,001–20000 (309–616 USD)	0.88 (0.66–1.15)	0.342			0.83 (0.63–1.08)	0.169		
>20,000 (>616 USD)	1.22 (0.92–1.62)	0.174			1.59 (1.21–2.09)	0.001		
Reimbursement scheme								
Government/state enterprises	Reference (1.00)				Reference (1.00)			
Universal coverage scheme	0.93 (0.62–1.38)	0.709			0.59 (0.41–0.86)	0.006		
Social security scheme	1.01 (0.67–1.51)	0.974			0.69 (0.47–1.01)	0.056		
Self-payment/others	0.97 (0.64–1.47)	0.892			0.65 (0.44–0.96)	0.030		
History of mental illness								
No	Reference (1.00)				Reference (1.00)			
Yes	0.78 (0.53–1.14)	0.205			0.66 (0.46–0.95)	0.026		
History of chronic NCDs ^‡^								
No	Reference (1.00)				Reference (1.00)			
Yes	1.17 (0.82–1.65)	0.383			1.79 (1.30–2.48)	<0.001		
Income loss during the COVID-19 pandemic								
No	Reference (1.00)				Reference (1.00)			
Yes	0.91 (0.72–1.16)	0.457			0.99 (0.78–1.24)	0.908		
Financial problems during the COVID-19 pandemic								
No	Reference (1.00)				Reference (1.00)			
Yes	0.92 (0.72–1.16)	0.463			1.53 (1.30–1.80)	0.362		
Information exposure during the COVID-19 pandemic								
<1 h/day	Reference (1.00)				Reference (1.00)		Reference (1.00)	
1–2 h/day	1.19 (0.92–1.55)	0.184			1.30 (1.01–1.67)	0.045	1.52 (1.05–2.21)	0.027
≥3 h/day	1.20 (0.87–1.66)	0.261			1.44 (1.06–1.96)	0.021	1.32 (0.84–2.07)	0.232
Confirmed cases in the community								
No	Reference (1.00)				Reference (1.00)			
Yes	1.17 (0.85–1.61)	0.341			1.46 (1.07–1.98)	0.016		
Not known	1.10 (0.83–1.46)	0.504			0.91 (0.69–1.21)	0.526		
Quarantine status								
Never	Reference (1.00)		Reference (1.00)		Reference (1.00)		Reference (1.00)	
Past	1.38 (1.07–1.78)	0.014	1.35 (0.99–1.84)	0.061	1.24 (0.97–1.59)	0.081	1.33 (0.92–1.93)	0.131
Current	1.73 (1.24–2.43)	0.001	2.08 (1.31–3.33)	0.002	1.34 (0.97–1.87)	0.076	1.75 (1.03–2.97)	0.039
Working from home								
No	Reference (1.00)				Reference (1.00)			
Yes	0.97 (0.73–1.28)	0.826			1.08 (0.82–1.41)	0.593		
Perceived social support								
Low perceived support	Reference (1.00)				Reference (1.00)			
Moderate perceived support	1.18 (0.71–1.96)	0.525			1.46 (0.92–2.32)	0.106		
High perceived support	1.83 (1.10–3.04)	0.020			1.98 (1.24–3.16)	0.004		
Resilient coping								
Low resilient copers	Reference (1.00)				Reference (1.00)			
Medium resilient copers	0.90 (0.64–1.27)	0.546			0.88 (0.63–1.23)	0.450		
High resilient copers	1.02 (0.73–1.43)	0.890			0.90 (0.65–1.24)	0.511		
Fear of COVID-19								
No/minimal	Reference (1.00)		Reference (1.00)		Reference (1.00)		Reference (1.00)	
Moderate	5.98 (3.4–10.42)	<0.001	3.70 (1.95–7.03)	<0.001	45.91 (11.13–189.40)	<0.001	20.71 (4.11–104.19)	<0.001
Severe	18.10 (10.04–32.61)	<0.001	6.24 (3.16–12.36)	<0.001	891.66 (215.11–3695.96)	<0.001	111.70 (21.90–569.63)	<0.001
Perceived risk of COVID-19 infection								
Low perceived risk	Reference (1.00)		Reference (1.00)		Reference (1.00)		Reference (1.00)	
Medium perceived risk	8.12 (6.06–10.90)	<0.001	7.78 (5.61–10.79)	<0.001	63.85 (27.90–146.14)	<0.001	60.15 (23.80–152.00)	<0.001
High perceived risk	52.43 (22.46–122.42)	<0.001	41.94 (17.56–100.15)	<0.001	3672.46 (1183.11–11399.61)	<0.001	2245.43 (667.25–7556.23)	<0.001

^†^ The effect estimates are presented weighted. ^‡^ To includes diabetes mellitus, hypertension, dyslipidemia, stroke and heart disease, chronic kidney disease, chronic lung disease, and cancer. ^§^ The currency exchange on the survey period was 1 USD = 32.5 Baht. Abbreviations: CI, confidence interval; COVID-19, coronavirus disease-2019; COVID-PSS, coronavirus disease-2019 Public Stigma Scale; NCDs, non-communicable diseases; OR, odds ratio.

## Data Availability

Data will be shared upon reasonable request and with permission according to the Health Outcomes and Mental Health Care Evaluation Survey Research Group (HOME-Survey) data release policy.

## Supplementary Materials

The following supporting information can be downloaded at: https://www.mdpi.com/article/10.3390/ijerph19116436/s1, Table S1: Participants Characteristics According to the Degree of Stigma towards COVID-19 Infection in Thailand; Table S2: Linear Regression Model Results of Factors Associated with Stigma towards COVID-19 Infection (n = 4004).
