# Supplementary material for: Disparities and Factors Associated with Coronavirus Disease-2019-Related Public Stigma: A Cross-Sectional Study in Thailand
Source: Int J Environ Res Public Health. 2022 May 25;19(11):6436. doi: 10.3390/ijerph19116436 (PMC9180735; doi:10.3390/ijerph19116436)
Supplement: Supplementary file 1 [file ijerph-19-06436-s001.zip › ijerph-1710632-supplementary.pdf]

# Online Supplementary Materials

**Disparities and Factors Associated with Coronavirus Disease-2019-Related Public Stigma: A Cross-Sectional Study in Thailand**

Chidchanok Ruengorn, Ratanaporn Awiphan, Chabaphai Phosuya, Yongyuth Ruanta, Kednapa Thavorn, Nahathai Wongpakaran, Tinakon Wongpakaran, Surapon Nochaiwong*; for the Health Outcomes and Mental Health Care Evaluation Survey Research Group (HOME-Survey)

***Correspondence and requests for materials:**

Surapon Nochaiwong, PharmD, Department of Pharmaceutical Care, Faculty of Pharmacy, Chiang Mai University, Chiang Mai 50200, Thailand, Phone: 66899973365, Fax: 6653222741, Email: surapon.nochaiwong@cmu.ac.th

# Supplementary Online Content

| | | |
|---|---|---|
| **Table S1** | Participants Characteristics According to the Degree of Stigma towards COVID-19 Infection in Thailand | S3 |
| **Table S2** | Linear Regression Model Results of Factors Associated with Stigma towards COVID-19 Infection (n=4004) | S6 |

**Table S1** Participants Characteristics According to the Degree of COVID-19-Related Public Stigma in Thailand

| Characteristics | Overall (n=4,004) | COVID-19-related public stigma | | | *P* Value |
|---|---|---|---|---|---|
| | | No/minimal: COVID-PSS ≤18 points (n=983) | Moderate: COVID-PSS 19 – 25 points (n=1,364) | High: COVID-PSS ≥26 points (n=1,657) | |
| COVID-PSS, mean (SD); range | 24.2 (7.6); 10–50 | 14.9 (2.6); 10–18 | 21.9 (2.0); 19–25 | 31.6 (4.8); 26–50 | <0.001 |
| Age, year, mean (SD); range | 29.1 (10.8); 18–79 | 27.6 (9.0); 18–65 | 27.9 (9.7); 18–79 | 30.8 (12.3); 18–73 | <0.001 |
| ≤30 | 2,659 (66.4) | 704 (71.6) | 953 (69.9) | 1,002 (60.5) | <0.001 |
| 31–50 | 1,088 (27.2) | 253 (25.8) | 355 (26.0) | 480 (29.0) | |
| ≥51 | 257 (6.4) | 26 (2.6) | 56 (4.1) | 175 (10.6) | |
| Sexual identity | | | | | |
| Female | 2,619 (65.4) | 673 (68.5) | 896 (65.7) | 1,050 (63.4) | 0.001 |
| Male | 1,231 (30.7) | 260 (26.4) | 415 (30.4) | 556 (33.5) | |
| Others | 154 (3.9) | 50 (5.1) | 53 (3.9) | 51 (3.1) | |
| Marital status | | | | | |
| Single | 3,208 (80.1) | 854 (86.9) | 1,162 (85.2) | 1,192 (71.9) | <0.001 |
| Married/domestic partnership | 693 (17.3) | 115 (11.7) | 170 (12.5) | 408 (24.6) | |
| Divorced/widowed/ separated | 103 (2.6) | 14 (1.4) | 32 (2.3) | 57 (3.4) | |
| Education level | | | | | |
| Illiterate/primary school/ junior high school | 127 (3.2) | 28 (2.8) | 41 (3.0) | 58 (3.5) | 0.459 |
| Senior high school/diploma/ high vocational | 1,893 (47.3) | 482 (49.0) | 654 (48.0) | 757 (45.7) | |
| Bachelor's degree/ higher education | 1,984 (49.5) | 473 (48.1) | 669 (49.0) | 842 (50.8) | |
| Occupation | | | | | |
| Unemployed/retried | 391 (9.8) | 95 (7.7) | 129 (9.5) | 167 (10.1) | 0.035 |
| Employed | 2,024 (50.5) | 480 (48.8) | 663 (48.6) | 881 (53.2) | |
| College student | 1,589 (39.7) | 408 (41.5) | 572 (41.9) | 609 (36.7) | |
| Religion | | | | | |
| Irreligion | 375 (9.4) | 143 (14.6) | 126 (9.2) | 106 (6.4) | <0.001 |
| Buddhist | 3,454 (86.3) | 787 (80.1) | 1,183 (86.7) | 1,484 (89.6) | |
| Christian/Muslim/Others | 175 (4.4) | 53 (5.4) | 55 (4.0) | 67 (4.0) | |

Data are expressed as the frequency (percentage) of patients, unless otherwise indicated.
Abbreviations: COVID-19, coronavirus disease-2019; COVID-PSS, coronavirus disease-2019 Public Stigma Scale; SD, standard deviation.

**Table S1** Participants Characteristics According to the Degree of COVID-19-Related Public Stigma in Thailand (Continued)

| **Characteristics** | **Overall (n=4,004)** | **COVID-19-related public stigma** | | | ***P* Value** |
|---|---|---|---|---|---|
| | | **No/minimal: COVID-PSS ≤18 points (n=983)** | **Moderate: COVID-PSS 19 – 25 points (n=1,364)** | **High: COVID-PSS ≥26 points (n=1,657)** | |
| Region of residence | | | | | |
| Capital city and its environs | 1,425 (35.6) | 412 (41.9) | 498 (36.5) | 515 (31.1) | <0.001 |
| Non-capital city and its environs | 2,579 (64.4) | 571 (58.1) | 866 (63.5) | 1,142 (68.9) | |
| Living status | | | | | |
| Alone | 576 (14.4) | 161 (16.4) | 203 (14.9) | 212 (12.8) | 0.023 |
| With family | 3,164 (79.0) | 745 (75.8) | 1,074 (78.7) | 1,345 (81.2) | |
| With others | 264 (6.6) | 77 (7.8) | 87 (6.4) | 100 (6.0) | |
| Person income, baht/month | | | | | |
| ≤10,000 | 1,905 (47.6) | 465 (47.3) | 654 (47.9) | 786 (47.4) | 0.001 |
| 10,001–20,000 | 1,054 (26.3) | 299 (30.4) | 357 (26.2) | 398 (24.0) | |
| >20,000 | 1,045 (26.1) | 219 (22.3) | 353 (25.9) | 473 (28.6) | |
| Reimbursement scheme | | | | | |
| Government/state enterprises | 539 (13.5) | 112 (11.4) | 157 (11.5) | 270 (16.3) | 0.002 |
| Universal coverage scheme | 1,329 (33.2) | 346 (35.2) | 466 (34.2) | 517 (31.2) | |
| Social security scheme | 1,161 (29.0) | 284 (28.9) | 402 (29.5) | 475 (28.7) | |
| Self-payment/others | 975 (24.3) | 241 (24.5) | 339 (24.8) | 395 (23.8) | |
| History of mental illness | 359 (9.0) | 108 (11.0) | 115 (8.4) | 136 (8.2) | 0.041 |
| History of chronic NCDs[†] | 599 (15.0) | 122 (12.4) | 177 (13.0) | 300 (18.1) | <0.001 |
| Income loss during the COVID-19 pandemic | 1,664 (41.6) | 398 (40.5) | 539 (39.5) | 727 (43.9) | 0.040 |
| Financial problems during the COVID-19 pandemic | 2,012 (50.2) | 485 (49.3) | 651 (47.7) | 876 (52.9) | 0.016 |

Data are expressed as the frequency (percentage) of patients, unless otherwise indicated.

[†]To includes diabetes mellitus, hypertension, dyslipidemia, stroke and heart disease, chronic kidney disease, chronic lung disease, and cancer.

Abbreviations: COVID-19, coronavirus disease-2019; COVID-PSS, coronavirus disease-2019 Public Stigma Scale; NCDs, non-communicable diseases.

**Table S1** Participants Characteristics According to the Degree of COVID-19-Related Public Stigma in Thailand (Continued)

| Characteristics | Overall (n=4,004) | COVID-19-related public stigma | | | *P* Value |
|---|---|---|---|---|---|
| | | No/minimal: COVID-PSS ≤18 points (n=983) | Moderate: COVID-PSS 19 – 25 points (n=1,364) | High: COVID-PSS ≥26 points (n=1,657) | |
| Information exposure during the COVID-19 pandemic | | | | | |
| <1 hour/day | 1,481 (37.0) | 408 (41.5) | 503 (36.9) | 570 (34.4) | 0.001 |
| 1–2 hours/day | 1,644 (41.1) | 391 (39.8) | 571 (41.9) | 682 (41.2) | |
| ≥3 hours/day | 879 (21.9) | 184 (18.7) | 290 (21.3) | 405 (24.4) | |
| Confirmed cases in the community | | | | | |
| No | 2,562 (64.0) | 637 (64.8) | 871 (63.9) | 1,054 (63.6) | 0.113 |
| Yes | 641 (16.0) | 136 (13.8) | 215 (15.8) | 290 (17.5) | |
| Not known | 801 (20.0) | 210 (21.4) | 278 (20.4) | 313 (18.9) | |
| Quarantine status | | | | | |
| Never | 1,781 (44.5) | 486 (49.4) | 567 (41.6) | 728 (43.9) | 0.004 |
| Past | 1,575 (39.3) | 359 (36.5) | 563 (41.3) | 653 (39.4) | |
| Current | 648 (16.2) | 138 (14.1) | 234 (17.1) | 276 (16.7) | |
| Working from home | 3,139 (78.4) | 774 (78.7) | 1,071 (78.5) | 1,294 (78.1) | 0.920 |
| Multidimensional scale of perceived social support, mean (SD); range | 59.1 (13.7); 12–84 | 58.0 (13.8); 12–84 | 59.7 (13.2); 12–84 | 59.2 (14.1); 12–84 | 0.016 |
| Low perceived support | 226 (5.6) | 59 (6.0) | 69 (5.1) | 98 (5.9) | <0.001 |
| Moderate perceived support | 1,833 (45.8) | 501 (51.0) | 574 (42.1) | 658 (45.8) | |
| High perceived support | 1,945 (48.6) | 423 (43.0) | 721 (52.9) | 801 (48.3) | |
| Brief resilient coping scale, mean (SD); range | 13.9 (3.1); 4–20 | 13.9 (3.1); 4–20 | 13.8 (3.1); 4–20 | 13.9 (3.0); 4–20 | 0.416 |
| Low resilient copers | 678 (16.9) | 165 (16.8) | 234 (17.2) | 279 (16.8) | 0.967 |
| Medium resilient copers | 1,570 (39.2) | 393 (40.0) | 525 (38.5) | 652 (39.4) | |
| High resilient copers | 1,756 (43.9) | 425 (43.2) | 605 (44.3) | 726 (43.8) | |

Data are expressed as the frequency (percentage) of patients, unless otherwise indicated.

Abbreviations: COVID-19, coronavirus disease-2019; COVID-PSS, coronavirus disease-2019 Public Stigma Scale; SD, standard deviation.

**Table S1** Participants Characteristics According to the Degree of COVID-19-Related Public Stigma in Thailand (Continued)

| **Characteristics** | **Overall (n=4,004)** | **COVID-19-related public stigma** | | | ***P* Value** |
|---|---|---|---|---|---|
| | | **No/minimal: COVID-PSS ≤18 points (n=983)** | **Moderate: COVID-PSS 19 – 25 points (n=1,364)** | **High: COVID-PSS ≥26 points (n=1,657)** | |
| Fear of COVID-19, mean (SD); range | 6.6 (1.8); 1–10 | 5.0 (1.5); 1–9 | 6.3 (1.4); 2–10 | 7.8 (1.4); 3–10 | <0.001 |
| No/minimal | 200 (5.0) | 169 (17.2) | 29 (2.1) | 2 (0.1) | <0.001 |
| Moderate | 1,698 (42.4) | 662 (67.3) | 754 (55.3) | 282 (17.0) | |
| Severe | 2,106 (52.6) | 152 (15.5) | 581 (42.6) | 1,373 (82.9) | |
| Perceived risk of COVID-19 infection, mean (SD); range | 5.5 (2.2); 2–10 | 3.3 (1.2); 2–10 | 5.1 (1.5); 2–10 | 7.2 (1.6); 2–10 | <0.001 |
| Low perceived risk | 767 (19.1) | 584 (59.4) | 171 (12.5) | 12 (0.7) | <0.001 |
| Medium perceived risk | 1,997 (49.9) | 385 (39.2) | 990 (72.6) | 622 (37.5) | |
| High perceived risk | 1,240 (31.0) | 14 (1.4) | 203 (14.9) | 1,023 (61.7) | |

Data are expressed as the frequency (percentage) of patients, unless otherwise indicated.

Abbreviations: COVID-19, coronavirus disease-2019; COVID-PSS, coronavirus disease-2019 Public Stigma Scale; SD, standard deviation.

**Table S2** Linear Regression Model Results of Factors Associated with COVID-19-Related Public Stigma (n=4,004)

| **Factors** | **Unadjusted beta coefficient (95% CI)**† | ***P* value** | **Adjusted beta coefficient (95% CI)**† | ***P* value** |
|---|---|---|---|---|
| Age, year | | | | |
| ≤30 | Reference (1.00) | | Reference (1.00) | |
| 31–50 | 0.49 (-0.24 to 1.22) | 0.188 | 0.98 (0.42 to 1.54) | 0.001 |
| ≥51 | 5.39 (3.99 to 6.79) | <0.001 | 3.03 (1.80 to 4.26) | <0.001 |
| Sexual identity | | | | |
| Female | Reference (1.00) | | Reference (1.00) | |
| Male | 1.09 (0.38 to 1.81) | 0.003 | 1.03 (0.54 to 1.52) | <0.001 |
| Others | -1.49 (-3.26 to 0.28) | 0.099 | 0.13 (-0.91 to 1.18) | 0.803 |
| Marital status | | | | |
| Single | Reference (1.00) | | Reference (1.00) | |
| Married/domestic partnership | 3.10 (2.16 to 4.04) | <0.001 | 0.98 (0.27 to 1.69) | 0.007 |
| Divorced/widowed/ separated | 3.83 (1.91 to 5.75) | <0.001 | 1.01 (-0.50 to 2.52) | 0.189 |
| Education level | | | | |
| Illiterate/primary school/ junior high school | Reference (1.00) | | Reference (1.00) | |
| Senior high school/diploma/ high vocational | -0.98 (-3.26 to 1.30) | 0.398 | … | … |
| Bachelor's degree/ higher education | -0.11 (-2.40 to 2.17) | 0.924 | … | … |
| Occupation | | | | |
| Unemployed/retried | Reference (1.00) | | Reference (1.00) | |
| Employed | 0.16 (-1.05 to 1.36) | 0.799 | … | … |
| College student | -0.45 (-1.66 to 0.76) | 0.466 | … | … |
| Religion | | | | |
| Irreligion | Reference (1.00) | | Reference (1.00) | |
| Buddhist | 2.45 (1.46 to 3.44) | <0.001 | 1.19 (0.54 to 1.83) | <0.001 |
| Christian/Muslim/Others | 2.06 (0.36 to 3.76) | 0.018 | 0.92 (-0.19 to 2.02) | 0.104 |

†The effect estimates are presented weighted.

Abbreviations: CI, confidence interval; COVID-19, coronavirus disease-2019; COVID-PSS, coronavirus disease-2019 Public Stigma Scale.

**Table S2** Linear Regression Model Results of Factors Associated with COVID-19-Related Public Stigma (n=4,004) (Continued)

| Factors | Unadjusted beta coefficient (95% CI)[†] | *P* value | Adjusted beta coefficient (95% CI)[†] | *P* value |
|---|---|---|---|---|
| Region of residence | | | | |
| Capital city and its environs | Reference (1.00) | | Reference (1.00) | |
| Non-capital city and its environs | 1.02 (0.46 to 1.59) | <0.001 | 0.40 (0.03 to 0.77) | 0.035 |
| Living status | | | | |
| Alone | Reference (1.00) | | Reference (1.00) | |
| With family | 1.10 (0.20 to 1.99) | 0.017 | … | … |
| With others | -0.69 (-2.12 to 0.73) | 0.340 | … | … |
| Person income, baht/month | | | | |
| ≤10,000 | Reference (1.00) | | Reference (1.00) | |
| 10,001 – 20,000 | -0.51 (-1.32 to 0.30) | 0.214 | … | … |
| >20,000 | 1.11 (0.34 to 1.87) | 0.005 | … | … |
| Reimbursement scheme | | | | |
| Government/state enterprises | Reference (1.00) | | Reference (1.00) | |
| Universal coverage scheme | -1.82 (-2.88 to -0.75) | 0.001 | … | … |
| Social security scheme | -1.40 (-2.49 to -0.31) | 0.012 | … | … |
| Self-payment/others | -1.71 (-2.77 to -0.64) | 0.002 | … | … |
| History of mental illness | | | | |
| No | Reference (1.00) | | Reference (1.00) | |
| Yes | -0.83 (-1.97 to 0.30) | 0.150 | … | … |
| History of chronic NCDs[‡] | | | | |
| No | Reference (1.00) | | Reference (1.00) | |
| Yes | 2.28 (1.34 to 3.23) | <0.001 | … | … |

[†]The effect estimates are presented weighted.

[‡]To includes diabetes mellitus, hypertension, dyslipidemia, stroke and heart disease, chronic kidney disease, chronic lung disease, and cancer.

Abbreviations: CI, confidence interval; COVID-19, coronavirus disease-2019; COVID-PSS, coronavirus disease-2019 Public Stigma Scale; NCD, non-communicable diseases.

**Table S2** Linear Regression Model Results of Factors Associated with COVID-19-Related Public Stigma (n=4,004) (Continued)

| **Factors** | **Unadjusted beta coefficient (95% CI)**† | ***P* value** | **Adjusted beta coefficient (95% CI)**† | ***P* value** |
|---|---|---|---|---|
| Income loss during the COVID-19 pandemic | | | | |
| No | Reference (1.00) | | Reference (1.00) | |
| Yes | 0.28 (-0.40 to 0.95) | 0.418 | … | … |
| Financial problems during the COVID-19 pandemic | | | | |
| No | Reference (1.00) | | Reference (1.00) | |
| Yes | 0.53 (-0.12 to 1.17) | 0.111 | … | … |
| Information exposure during the COVID-19 pandemic | | | | |
| <1 hour/day | Reference (1.00) | | Reference (1.00) | |
| 1–2 hours/day | 0.54 (-0.17 to 1.25) | 0.136 | … | … |
| ≥3 hours/day | 1.33 (0.41 to 2.26) | 0.005 | … | … |
| Confirmed cases in the community | | | | |
| No | Reference (1.00) | | Reference (1.00) | |
| Yes | 0.45 (-0.36 to 1.26) | 0.280 | … | … |
| Not known | -0.47 (-1.25 to 0.32) | 0.244 | … | … |
| Quarantine status | | | | |
| Never | Reference (1.00) | | Reference (1.00) | |
| Past | 0.74 (0.02 to 1.46) | 0.043 | 0.79 (0.32 to 1.27) | 0.001 |
| Current | 0.56 (-0.35 to 1.48) | 0.228 | 0.64 (0.01 to 1.27) | 0.046 |
| Working from home | | | | |
| No | Reference (1.00) | | Reference (1.00) | |
| Yes | -0.17 (-0.98 to 0.65) | 0.688 | … | … |
| Perceived social support | | | | |
| Low perceived support | Reference (1.00) | | Reference (1.00) | |
| Moderate perceived support | 0.97 (-0.34 to 2.29) | 0.147 | … | … |
| High perceived support | 1.92 (0.61 to 3.22) | 0.004 | … | … |

†The effect estimates are presented weighted.

Abbreviations: CI, confidence interval; COVID-19, coronavirus disease-2019; COVID-PSS, coronavirus disease-2019 Public Stigma Scale.

**Table S2** Linear Regression Model Results of Factors Associated with COVID-19-Related Public Stigma (n=4,004) (Continued)

| **Factors** | **Unadjusted beta coefficient (95% CI)†** | ***P* value** | **Adjusted beta coefficient (95% CI)†** | ***P* value** |
|---|---|---|---|---|
| Resilient coping | | | | |
| Low resilient copers | Reference (1.00) | | Reference (1.00) | |
| Medium resilient copers | -1.01 (-2.03 to 0.01) | 0.052 | -0.76 (-1.46 to -0.05) | 0.036 |
| High resilient copers | -1.00 (-1.97 to -0.03) | 0.043 | -0.82 (-1.51 to -0.14) | 0.019 |
| Fear of COVID-19 | | | | |
| No/minimal | Reference (1.00) | | Reference (1.00) | |
| Moderate | 5.01 (4.23 to 5.79) | <0.001 | 2.06 (1.35 to 2.76) | <0.001 |
| Severe | 12.76 (11.94 to 13.57) | <0.001 | 5.11 (4.30 to 5.92) | <0.001 |
| Perceived risk of COVID-19 infection | | | | |
| Low perceived risk | Reference (1.00) | | Reference (1.00) | |
| Medium perceived risk | 6.95 (6.43 to 7.47) | <0.001 | 5.60 (5.05 to 6.15) | <0.001 |
| High perceived risk | 15.11 (14.46 to 15.76) | <0.001 | 12.21 (11.47 to 12.96) | <0.001 |

†The effect estimates are presented weighted.

Abbreviations: CI, confidence interval; COVID-19, coronavirus disease-2019; COVID-PSS, coronavirus disease-2019 Public Stigma Scale.
